# Anti-cancer effects of *Gynostemma pentaphyllum* (Thunb.) Makino (*Jiaogulan*)

**DOI:** 10.1186/s13020-016-0114-9

**Published:** 2016-09-27

**Authors:** Yantao Li, Wanjun Lin, Jiajun Huang, Ying Xie, Wenzhe Ma

**Affiliations:** State Key Laboratory of Quality Research in Chinese Medicine, Macau University of Science and Technology, Taipa, Macao, China

## Abstract

*Gynostemma pentaphyllum* (Thunb.) Makino (GpM) (*Jiaogulan*) has been widely used in Chinese medicine for the treatment of several diseases, including hepatitis, diabetes and cardiovascular disease. Furthermore, GpM has recently been shown to exhibit potent anti-cancer activities. In this review, we have summarized recent research progress on the anti-cancer activities and mechanisms of action of GpM, as well as determining the material basis for the anti-cancer effects of GpM by searching the PubMed, Web of Science and China National Knowledge Infrastructure databases. The content of this review is based on studies reported in the literature pertaining to the chemical components or anti-cancer effects of GpM up until the beginning of August, 2016. This search of the literature revealed that more than 230 compounds have been isolated from GpM, and that most of these compounds (189) were saponins, which are also known as gypenosides. All of the remaining compounds were classified as sterols, flavonoids or polysaccharides. Various extracts and fractions of GpM, as well as numerous pure compounds isolated from this herb exhibited inhibitory activity towards the proliferation of cancer cells in vitro and in vivo. Furthermore, the results of several clinical studies have shown that GpM formula could have potential curative effects on cancer. Multiple mechanisms of action have been proposed regarding the anti-cancer activities of GpM, including cell cycle arrest, apoptosis, inhibition of invasion and metastasis, inhibition of glycolysis and immunomodulating activities.

**Electronic supplementary material:**

The online version of this article (doi:10.1186/s13020-016-0114-9) contains supplementary material, which is available to authorized users.

## Background

Cancer is the world’s leading cause of death, accounting for 8.2 million deaths in 2012, and it is expected that the annual number of global cancer cases will rise from 14 million in 2012 to 22 million within the next two decades [1]. The isolation and evaluation of anti-cancer agents and lead compounds from natural resources represents a traditional and effective approach for the development of new drugs for the treatment of cancer [2, 3], as exemplified by Paclitaxel, which was derived from *Taxus brevifolia* [3, 4].

*Gynostemma pentaphyllum* (Thunb.) Makino (GpM) (*Jiaogulan*) has been widely used in Chinese medicine for the treatment of various diseases, including hepatitis, diabetes and cardiovascular disease. Modern medical research has shown that GpM exhibits a variety of pharmacological properties, including anti-inflammatory [5–8], antioxidative [9–13], lipid metabolism regulatory [14–18], antiproliferative [19–22], neuroprotective [23, 24] and anxiolytic activities [25–27]. GpM has consequently been widely used for the treatment of hepatitis [15, 28–30], diabetes [11, 30–32], cardiovascular disease [33–35] and cancer [20, 23, 36, 37]. GpM is also widely used as a health supplement in beverages, biscuits, noodles, face washes and bath oils [38–41].

We have conducted a comprehensive review of the literature associated with GpM to provide a summary of recent research towards the anti-cancer activities and mechanisms of action of GpM. We have also searched the PubMed, Web of Science and China National Knowledge Infrastructure (CNKI) databases to identify the material basis for the anti-cancer effects of GpM.

### Literature search strategy and exclusion criteria

Our literature search covered all of the records in the PubMed, Web of Science Core Collection and CNKI databases up until the beginning of August, 2016. All of the studies included in this review reported on either the chemical components or the anti-cancer effects of GpM. The following search strategy was used to search PubMed with the “All Fields” option and the Web of Science Core Collection in the “Topic” field (Table 1). Studies were excluded if they were: (a) duplicated; (b) not pertinent to the chemical components or the anti-cancer effects of GpM; or (c) not full-text journal articles.

**Table 1:** Strategy in searching PubMed and Web of Science

Step	Search terms	Citations reviewed	
		PubMed	Web of Science
1	*Gynostemma pentaphyllum*	280	314
2	Jiaogulan	284	22
3	Gypenoside	137	96
4	Constituents	58,058	137,552
5	Composition	328,924	780,071
6	Components	471,244	1,371,871
7	Tumor	3,285,652	983,570
8	Cancer	3,336,622	1,467,455
9	Carcinoma	797,702	521,059
10	1 or 2 or 3	322	364
11	4 or 5 or 6	821,820	2,163,770
12	7 or 8 or 9	3,699,522	2,049,723
13	10 and 11	66	109
14	10 and 12	81	83
15	13 or 14	130	162

Clinical studies were not excluded from the searches conducted using the PubMed and Web of Science database; however, no records involving clinical trials were found in either of these databases. For this reason, we searched the CNKI database for clinical studies pertaining to the use of GpM for the treatment of cancer. The following search strategy was used to search the CNKI database using the “Abstract” field (Table 2). Studies were excluded from the results if they were: (a) not pertinent to the clinical anti-cancer effects of GpM; (b) review articles; or (c) not full-text journal articles.

**Table 2:** Strategy in searching CNKI

Step	Search terms	Citations reviewed
1	Jiaogulan	2876
2	Zhongliu	767,939
3	Linchuang	3,366,530
4	1, 2 and 3	43

**Fig. 1:**
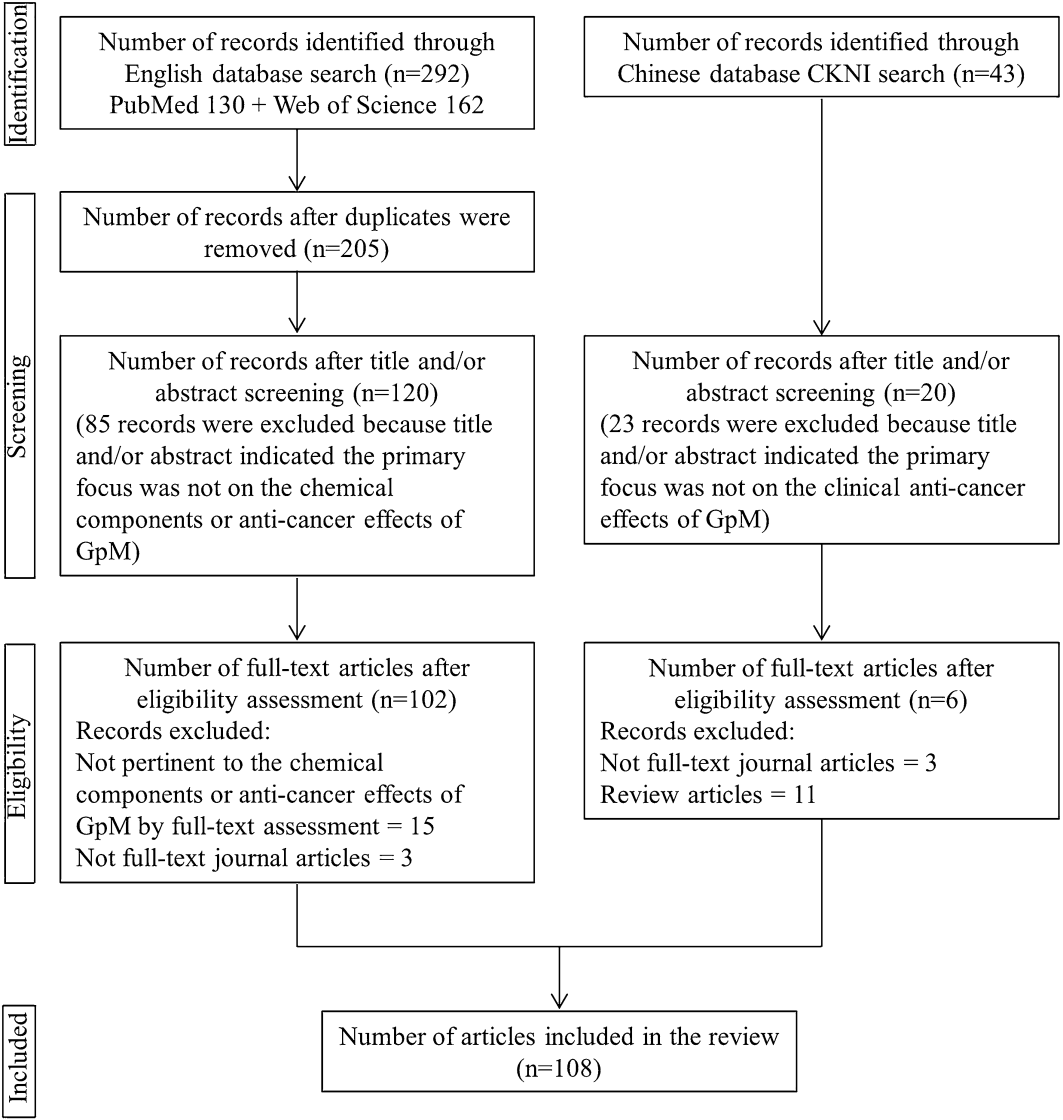
Flowchart indicating the search strategies and processes used in this study

Two reviewers independently searched the databases and screened all of the articles for their eligibility. The search strategies and selection processes used by the reviewers are shown in Fig. 1. In this way, we identified 108 articles, which have been included in this review.

### Chemical components of GpM

Over 230 compounds have been identified as being derived from GpM and can be grouped according to their chemical structures into saponins, sterols, flavonoids, polysaccharides and several other compound classes.

### Saponins

One hundred and eighty-nice saponins, which are also known as gypenosides (Gyps), have been isolated from GpM and fully characterized using spectroscopic methods [42–45]. At the 23rd meeting of the Japanese society of pharmacognosy, Nagai et al. [46] reported the isolation of two dammarane-type saponins, including panoxadiol and 2α-OH-panoxadiol, from the hydrolysate of Gyps. The research groups of Takemotoet [47–50] and Yoshikawa [51, 52] subsequently reported the isolation and characterization of many other Gyps from GpM. Gyps are the major components of GpM, and mainly consist of triterpenoid saponins, which can be purified from ethyl acetate or n-butanol extracts [53]. One hundred and sixty-five of the 189 Gyps reported to date have been grouped into 12 classes based on the nature of their aglycone moiety [43]. However, the remaining 24 Gyps [44, 45, 54–60] do not belong to any of these classes. The general structure of Gyps is shown in Table 3, and shows that these compounds consist of a hydrophobic sapogenin moiety (where R_1_ = hydrocarbon chain with or without double bond(s), cyclopentane, oxacyclopentane or some other group) and a hydrophilic sugar group (where R_4_ = glucose, rhamnose or xylose).

**Table 3:** Structures and in vitro anti-cancer activity of identified GpM components


Compd R_1_		R_2_	R_3_	R_4_	R_5_	R_6_	Anti-cancer activity^a^	Reference
1		H	H		CH_3_	H	47.12 (HL-60)	[122]
2		H	H		CH_3_	H	45.50 (HL-60)	[122]
3		H	H		CH_3_	H	HL-60, Colon 205, Du145, GC-7901, BEL-7402	[85, 86]
4		H	H		CH_3_	H	HL-60, Colon 205, Du145, GC-7901, BEL-7402	[85, 86]
5		H	H		CHO	H	HL-60, Colon 205, Du145, GC-7901, BEL-7402	[85, 86]
6		H	H		CHO	H	HL-60, Colon 205, Du145, GC-7901, BEL-7402	[85, 86]
7		CH_3_	H		CHO	H	HL-60, Colon 205, Du145, GC-7901, BEL-7402	[85, 86]
8		CH_3_	H		CHO	H	HL-60, Colon 205, Du145, GC-7901, BEL-7402	[85, 86]
9		CH_3_	H		CH_3_	H	HL-60, Colon 205, Du145, GC-7901, BEL-7402	[85, 86]
10		CH_3_	H		CH_3_	OH	67.66 ± 3.36 (HL-60), 18.45 ± 0.93 (MCF-7), 34.95 ± 0.93(HT-29), 20.97 ± 1.49 (A549), 27.68 ± 1.58 (SK-OV-3)	[53]
11		CH_3_	H		CH_3_	=O	>109.2 (HL-60), 42.81 ± 3.60 (MCF-7), 22.06 ± 2.18 (HT-29), 31.45 ± 2.62 (A549), 30.25 ± 1.53 (SK-OV-3)	[53]
12		CH_3_	H		CH_3_	=O	>107.6 (HL-60), 23.03 ± 1.40 (MCF-7), 46.30 ± 1.08 (HT-29), 21.09 ± 1.18 (A549), 35.62 ± 0.97 (SK-OV-3)	[53]
13		CH_3_	H		CH_3_	=O	76.63 ± 2.98 (HL-60), 23.62 ± 1.02 (MCF-7), 39.34 ± 1.02 (HT-29), 19.90 ± 1.40 (A549), 19.90 ± 1.49 (SK-OV-3)	[53]
14		H	H		CH_3_	H	7.44 (HL-60), 27.80 (Colon 205), 24.12 (Du145)	[127]
15		H	H	H	CH_3_	H	3.90 (MDA-MB-435)	[128]
16		H	H	H	CH_3_	H	0.05 ± 0.01 (A549), 0.25 ± 0.07 (U87)	[129]
17		H	OH	H	CH_3_	OH	12.54 ± 0.53 (A549)	[130]
18		H	OH		CH_3_	OH	34.94 ± 4.23 (A549)	[130]
19		H	H	H	CH_3_	H	40 ± 0.7 (HepG2)	[44]
20		H	H	H	CH_3_	OH	38 ± 0.5 (HepG2)	[44]
21		H	H	H	CH_3_	H	41.89 (HCT116), 20.94 (HT-29), 32.61 (MCF-7)	[55]
22		H	H	H	CH_3_	H	41.40 (HCT116), 19.00 (HT-29), 28.82 (MCF-7)	[55]
23		H	H		CHO	H	32.00 ± 1.24 (HepG2)	[56]
24		H	H		CH_3_	H	21.38 ± 1.06 (HepG2)	[56]
25		H	OH		CH_3_	OH	74.3 ± 1.9 (A549)	[59]
26							18.41 (HT-29), 4.46 (MCF-7), 9.39 ± 0.9 (DI145), 6.93 ± 0.5 (22RV-1)	[54, 55]
27							20.38 (HT-29), 13.51 (MCF-7)	[55]
28							16.14 (HT-29), 8.84 (MCF-7)	[55]
Flavonoid fraction							33.3 (PC-3)	[21]
Carotenoid fraction							1.6 (Hep3B)	[84]
Chlorophyll fraction							57.5 (Hep3B)	[84]
Nonpolar fraction							38.02 ± 2.98 (MDA-MB-453), 31.62 ± 1.76 (HCT116), 35.48 ± 3.81 (LNCaP), 35.48 ± 6.45 (MCF7)	[22]
Gypenosides							47.6 (Hep3B), 39.3 (PC-3), 30.6 (A549) HL-60, MCF-7, HT-29, Colon 205, Du145, MDA-MB-435, U87, A549, SK-OV-3, HepG2, SGC-7901, BEL-7402, Huh-7, HA22T, SW620, Eca-109, SAS, L1210, WEHI-3, SW-480, KB/VCR, MCF-7/ADR	[19, 21, 36, 44, 85–87, 89–91, 105–109, 111, 112, 114, 115, 119, 121, 131, 132]
Polysaccharide							65.4 (B16), HT-29, B16, Hela, SW-1116, HepG2	[44, 87, 88, 119]
Ethanolic extract							C6, HT-59	[113, 133]

^a^The anti-cancer activities, IC_50_ (μg/ml), of components of GpM are expressed as mean ± SD following cell line names in bracket. Only the mean value is listed if there is no SD value available and only cancer cell line name is listed if no IC_50_ data is available. The unit of compound 10, 11, 12, 13, 16, 21, 22, 26, 27 and 28 are transformed based on molecular weight. This table also presents 30 cancer cell lines whose proliferation could be inhibited by GpM, which indicates that GpM exerts broad spectrum anti-cancer activities

### Sterols

Sterols are composed of 17 carbon atoms across four rings, i.e., three 6-carbon rings and a single 5-carbon ring, with a side chain extending from C17 containing nine or ten carbon atoms (Additional file 1: Table S1). Eighteen sterols were isolated from GpM and fully characterized using a unique method from 1986 to 1990 [61–67]. Briefly, GpM was extracted with CH_2_Cl_2_, and the extracted lipids were saponified with 5 % KOH in MeOH. After purification by column chromatography over silica gel, the sterol mixture was acetylated, crystallized and characterized using spectroscopic methods. This process resulted in the isolation of sterols with ergostane, cholestane and stigmastane skeletons. The structures of these 18 sterols are shown in Additional file 1: Table S1. These compounds contained one double bond between C5–C6, C7–C8 or C9–C11, with R_2_ = H or CH_3_ and R_1_ = hydrocarbon chain with 10 carbons, and one double bond or one alkynyl group.

### Polysaccharides

Polysaccharides are major components of GpM, where they are typically conjugated with proteins [68]. The molecular weight of the polysaccharides found in GpM varies from 9000 to 33,000 Da [69]. Several different kinds of polysaccharides have been found in GpM, and the molar ratios of the monosaccharide components of these systems have been reported to vary considerably. For instance, the neutral polysaccharide fraction CGPP mainly consists of mannose, glucose, arabinose, rhamnose, galactose and glucuronic acid with molar ratios of 2.0:2.2:1.3:2.2:1.2:2.5 [20]. Another polysaccharide fraction (NaCl eluted fraction of crude polysaccharides from GpM by DEAE-Sepharose CL-6B chromatography, GMC) consisted of glucose, galactose, mannose and fructose with the molar ratios of 1:2.17:1.25:1.02 [70]. Furthermore, the water-soluble GpM polysaccharide fraction GP-I contains glucose, galactose, mannose, rhamnose and arabinose with molar ratios of 5.3:4.2:3.0:0.7:0.8 [69]. Based on the differences in the possible arrangements of the monosaccharides, various polysaccharides have been isolated from GpM [71].

### Flavonoids

Flavonoids are an important class of polyphenol compounds that are widely distributed in fruits and vegetables, where they usually exist in their glycosidic form [72]. In terms of their general structure, flavonoids consist of a 15-carbon skeleton, containing two phenyl rings (A and B) and a heterocyclic ring (C). The carbon structure of these compounds is usually abbreviated as C6–C3–C6. Several flavonoids have been isolated from GpM, including quercetin, rutin, ombuoside [73], ombuin [74], isorhamnetin-3-*O*-rutinoside, isorhamnetin [75], quercetin-di-(rhamno)-hexoside, quercetin-rhamno-hexoside, kaempferol-rhamno-hexoside and kaempferol-3-*O*-rutinoside [76], and the structures of these flavonoids are shown in Additional file 1: Table S2.

### Other components of GpM

GpM contains various trace elements (e.g., Cu, Fe, Zn, Mn, Co, Ni, Se, Mo and Sr) [77], 18 amino acids [78] (including eight essential amino acids) and various vitamins and proteins, but the relative amounts of these components vary considerably across the different parts of the GpM plant (i.e., leaf, stem and subterranean stem) [78]. Malonic acid [74], benzyl-*O*-β-d-glucopyranoside [79], lutein, vomifoliol, palmitic acid [80], linolenic acids [81, 82] and carrot glycosides [83] have also been isolated from GpM. Furthermore, Tsai et al. [84] reported the isolation of numerous carotenoids and chlorophylls from the carotenoid and chlorophyll fractions of GpM, respectively.

### Anti-cancer activities of GpM

#### In vitro anti-cancer activities of GpM

The in vitro antiproliferative activities of some of the pure compounds and extracts isolated from GpM have been widely reported and the details of these materials are summarized in Table 3. Shi et al. [85] obtained four dammarane-type triterpene saponins (compounds **3–6**) from the aerial parts of GpM, which exhibited moderate cytotoxic activities in vitro against several human cancer cell lines, including HL-60 (human promyelocytic leukemia cells), Colon 205 (human colon cancer cells) and Du145 (human prostate carcinoma cells) cells. Yin et al. [86] isolated nine dammarane saponins from the methanol extract of the aerial part of GpM, and found that compounds **7**, **8** and **9** exhibited inhibitory activities towards the growth of SGC-7901 (stomach cancer cells) and BEL-74020 (hepatocellular carcinoma cells) at a concentration of 100 μM with percentage inhibition values of 21, 93 and 8 %, and 77, 92 and 40 %, respectively.

Almost all of the compounds and extracts isolated from GpM to date have be reported to exhibit noticeable antiproliferative activities with IC_50_ values ranging from 0.05 to 74.3 μg/mL (Table 3). Compound **16** exhibited potent antiproliferative activities against A549 human lung cancer cells and U87 glioblastoma cells with IC_50_ values of 0.05 and 0.25 μg/mL, respectively. Compound **15** showed antiproliferative activity against MDA-MB-435 human breast cancer cells with an IC_50_ value of 3.90 μg/mL, whereas the carotenoid fraction of GpM exhibited the strongest activities of all of the reported extracts with an IC_50_ value of 1.6 μg/mL against Hep3B human hepatocellular carcinoma cells.

The hydrolysates of the extracts of GpM have also been reported to exhibit anti-cancer activities, together with several other derivatives of the natural products found in GpM. For example, Chen et al. [87] reported the synthesis of four sulfated derivatives of GPP2, which is a native polysaccharide isolated from GpM. One of the sulfated derivatives prepared by Chen (GPP2-s4) inhibited the growth of HepG2 human hepatocellular carcinoma cells by 46.4 ± 2.8 % at a concentration of 2000 μg/mL. Compared with GPP2, all four sulfated derivatives exhibited stronger antiproliferative activities against HeLa cervical cancer cells at concentrations as low as 100 μg/mL. GP-B1, which is an acidic polysaccharide derived from GpM, significantly inhibited the growth of B16 melanoma cells with an IC_50_ of 65.4 μg/mL with very little cytotoxicity against normal cells [88]. Moreover, GP-B1 not only significantly inhibited the growth of cancer cells, but also improved cellular immune response by increasing levels of tumor necrosis factor-α (TNF-α), interferon-γ (IFN-γ), interleukin-10 (IL-10) and interleukin-12 (IL-12) observed in the serum of melanoma-B16-bearing mice [88].

#### In vivo anti-cancer activities of GpM

The in vivo anti-cancer activities of GpM are summarized in Table 4. Gyps led to significant reductions in the size of solid tumors in nude mice injected with SAS oral cancer cells [89]. Gyps also promoted the survival of mice xenografted with WEHI-3 leukemia cells, which was accompanied by an increase in the number of megakaryocytes and reduced spleen weight in these animals, indicating an enhanced immune response [90]. Similar anti-cancer activities have also been reported for Gyps in another leukemia mouse model [91]. The intraperitoneal treatment of tumor-bearing mice with Gyps (5 or 20 mg/kg/day) for 4 weeks led to considerable decreases in the size and weight of their tumors without altering their body weight. Gyps also strongly suppressed tumor growth in mice bearing advanced S180 sarcoma, which was associated with an increase in the ratio of tumor necrosis area to tumor total area and lymphocyte/macrophage infiltration into the peripheral areas of tumors. This effect also led to an increase in the weight of the spleens of these animals, as well as increases in the quantity and size of their splenic white pulp [92]. Gyps enhanced the anti-cancer effects of 5-fluorouracil in colorectal cancer cells and xenografts [93]. Gyps have also been reported to inhibit tumorigenesis in a transgenic mouse models of cancer, such as the Apc^*Min/*+^ mouse model of intestinal neoplasia [94, 95]. Moreover, rats fed with a standardized extract of GpM did not show any mortal or toxic effects, highlighting the good safety profile of this material [96].

**Table 4:** In vivo anti-cancer activity of identified GpM components

Component	Animal model	Anti-cancer activity^a^	Reference
Gypenosides	Nude mice: xenografted with human oral cancer SAS cells	65.76 % (tumor size, 20 mg/kg for 28 days)	[89]
Gypenosides	BALB/c mice: injected with human leukemia WEHI-3 cells	150 % (survival rate, 2 mg/kg for 2 weeks) 175 % (survival rate, 4 mg/kg for 2 weeks)	[90]
Gypenosides	Nude mice: xenografted with human leukemia HL-60 cells	44 % (tumor size, 20 mg/kg for 28 days)	[91]
Gypenosides	BALB/c mice: xenografted with murine S180 sarcoma cells	39.57 % (tumor size, 30 mg/kg for 4 days)	[92]
Gypenosides	BALB/c mice: xenografted with murine colorectal cancer CT-26 cells	75 % (tumor size, 25 mg/kg for 19 days) 55 % (tumor size, 50 mg/kg for 19 days) 26 % (tumor size, 50 mg/kg + 5 mg/kg 5-Fu for 19 days)	[93]
Gypenosides	*Apc* ^*Min/*+^ mice: intestinal neoplasia model	66.06 % (polyps number, 500 mg/kg for 4 weeks) 59.92 % (polyps number, 750 mg/kg for 4 weeks)	[94]
Gypenosides	*Apc* ^*Min/*+^ mice: intestinal neoplasia model	59.32 % (polyps number, 500 mg/kg for 8 weeks)	[95]
Polysaccharide	BALB/c mice: xenografted with murine S180 sarcoma cells	62.77 % (tumor size, 100 mg/kg for 14 days) 59.24 % (tumor size, 200 mg/kg for 14 days)	[97]
Polysaccharide	ICR mice: xenografted with mouse hepatoma H22 cells	62.89 % (tumor size, 50 mg/kg for 10 days) 49.22 % (tumor size, 200 mg/kg for 10 days)	[20]

^a^The anti-cancer activities of components of GpM are expressed as the percentage of control (readout, dose)

A polysaccharide from GpM inhibited the development of transplanted S180 sarcoma in a dose-dependent manner and increased the phagocytosis of macrophages, as well as increasing the production of NO, IL-1β and TNF-α from the peritoneal macrophages [97]. The neutral polysaccharide fraction CGPP inhibited the growth of H22 hepatocarcinoma cells transplanted into ICR mice [20]. CGPP treatment also led to improvements in the body weight, spleen/thymus index and degree of splenocyte proliferation in tumor-bearing mice [20]. Furthermore, CGPP treatment led to considerable increases in the levels of cytokines, such as IL-2, TNF-α and IFN-γ in tumor-bearing mice, as well as increases in the activity of natural killer (NK) cells and cytotoxic T lymphocytes (CTL) [20]. The tumor inhibitory and immunoregulatory effects of CGPP greatly increased the life span of H22 ascites in tumor-bearing mice [20].

### Clinical anti-cancer studies on GpM

A clinical study was conducted in 1993 involving 59 patients with advanced malignant tumors to assess the effects of GpM [98]. The results revealed that patient treated with a GpM formula showed cancer relapse and metastasis rates of 11.9 and 8.5 %, respectively, compared with values of 72.4 and 55.2 % in the control group. The results of this study also revealed that the T lymphocyte transformation rate and acid α-naphthyl acetate esterase (ANAE+) activity increased by 8.2 % following GpM treatment [98]. The results of a separate 5-year observational study also showed that the treatment of cancer patients with GpM formula led to significant reductions in cancer relapse and metastasis rates, as well as reduced mortality and improved immune function in these patients [99]. GpM has also been reported to enhance NK cell activity in breast cancer patients [100], and improve the immune function of cancer patients after chemotherapy, as demonstrated by increased T lymphocyte transformation rate and decreased IgG and IgM levels [101]. Furthermore, GpM enhanced the immunological function of lung cancer patients after chemotherapy [102]. The results of a recent study [103] demonstrated that GpM formula can work in synergy with chemotherapy reagents. The clinical uses of GpM are summarized in Table 5.

**Table 5:** Clinical uses of GpM

Component	Patient tumor type	Anti-cancer activity^a^	Reference
GpM formula	Multiple types	16.44 % (relapse rate) 15.40 % (metastasis rate)	[98]
GpM formula	Multiple types	14.23 % (relapse and metastasis rate)	[99]
GpM formula	Breast cancer	129.56 % (NK cell activity)	[100]
GpM formula	Multiple types	157 % (T lymphocyte transformation rate) 78.4 % (IgG levels) 75.1 % (IgA levels) 59.9 % (IgM levels)	[101]
GpM formula	Lung cancer	128 % (curative rate)	[102]
GpM formula	Middle-late gastric cancer	163 % (short term curative rate) 140 % (quality of life)	[103]

^a^The anti-cancer activities of components of GpM are expressed as the percentage of control (readout)

### Mechanisms of action

**Fig. 2:**
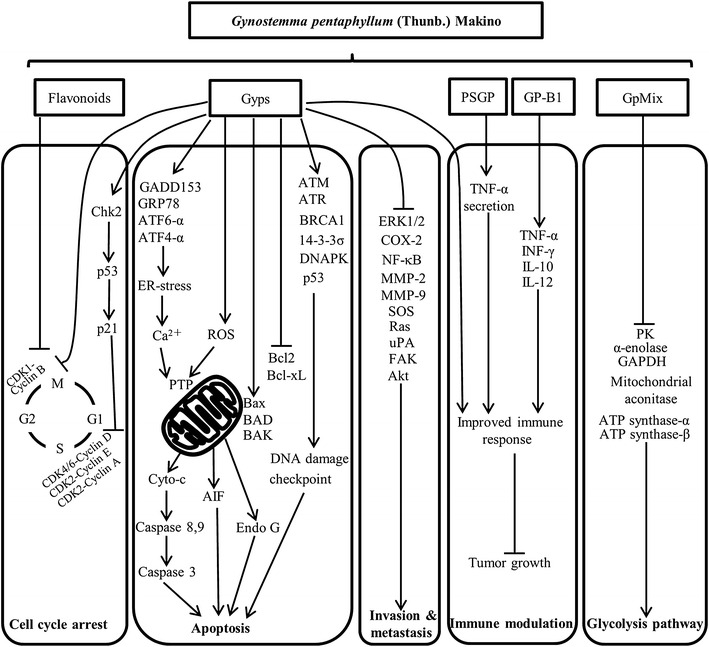
Mechanisms of action for the anti-cancer activities of GpM

Multiple mechanisms of action have been proposed regarding the anti-cancer activities of GpM, including cell cycle arrest, apoptosis induction, inhibition of invasion and metastasis, glycolysis inhibition and immunomodulation (Fig. 2).

### Cell cycle arrest

Gyps induced cell cycle arrest at the G0/G1 phase SAS human oral cancer cells [89], WEHI-3 leukemia cells [90], A549 human lung adenocarcinoma cells [104], HL-60 human myeloid leukemia cells [91] and Colo 205 human colon cancer cells [105]. Gyps also induced cell cycle arrest by modulating the expression of several cell cycle regulatory proteins, including cyclin-dependent kinase 2 (CDK2), cyclin-dependent kinase 4 (CDK4) and cyclin-dependent kinase 6 (CDK6) [21, 106]. The treatment of SCC-4 human tongue cancer cells with Gyps induced checkpoint kinase 2 (Chk2) expression. This effect subsequently led to the upregulation of p53 and its targets p21 and p16, which led to decreased levels of cyclin D and cyclin E and G0/G1 cell cycle arrest [106]. The treatment of PC-3 human prostate carcinoma cells with flavonoids and saponins isolated from GpM led to cell cycle arrest in the S and G2/M phases in both cases by modulating the expression of cyclins [21]. Furthermore, A549 cells treated with flavonoids from GpM went into cell cycle arrest in the S and G2/M phases, and showed upregulated levels of Cyclin A, Cyclin B, p21 and p53 [24].

### Induction of apoptosis

A large number of studies have shown that GpM exerts its anti-cancer activities by inducing cellular apoptosis through various signaling pathways. Gyps downregulated the anti-apoptotic proteins Bcl-2 and Bcl-xL, and upregulated the pro-apoptotic proteins Bax, Bad and Bak, thereby activating the formation of Bax/Bak pores on the outer mitochondrial membrane [89–91, 107, 108]. Bax/Bak pores allow for the release of cytochrome c and other pro-apoptotic proteins into the cytosol, leading to the activation of initiator caspases-8 and -9, followed by the cleavage of effector caspase-3, which ultimately triggers apoptosis [104–106]. The formation of Bax/Bak pores following Gyps treatment also led to the release of apoptosis inducing factor (AIF) and endonuclease G (EndoG) from the mitochondria [89, 106], following DNA fragmentation and chromatin condensation.

Gyps also induced the production of reactive oxygen species (ROS) [36, 89, 105, 106, 108, 109] and led to increased intracellular Ca^2+^ concentrations [89–91, 105, 106, 108]. ROS and Ca^2+^ are both well-studied modulators of the permeability transition pores located on the inner mitochondrial membrane. The opening of these pores leads to an influx of solutes and water into the mitochondrial matrix, causing the outer mitochondrial matrix to swell and rupture, which leads to the release of cytochrome c and apoptosis [89–91, 105–107]. Gyps treatment led to increased levels of DNA-damage-inducible transcript 3 (GADD153), glucose-regulated protein (GRP78), activating transcription factor 6 alpha (ATF6-α) and activating transcription factor 4 alpha (ATF4-α). These increases resulted in endoplasmic reticulum (ER) stress, which could result in the release of Ca^2+^ from the ER [89–91, 106]. Moreover, Sun et al. [110] reported increased store-operated Ca^2+^ entry as another mechanism of action for the activity of Gyps.

Furthermore, Gyps induced dose-dependent DNA damage in SAS cells and reduced the expression of several DNA repair genes, including ataxia telangiectasia mutated, ataxia-telangiectasia and Rad3-related, breast cancer gene 1, 14-3-3σ, DNA-dependent serine/threonine protein kinase and p53, in a time-dependent manner. In this way, Gyps treatment stalled the DNA damage repair process, forcing the cells to undergo apoptosis [109, 111].

Several other components and fractions of GpM have also been reported to induce apoptosis. For instance, flavonoids [21] and a water extract [112] from GpM induced apoptosis in tumor cells via the regulation of the Bcl-2 protein family. Furthermore, an ethanolic extract from GpM selectively shifted the intracellular H_2_O_2_ concentration to toxic levels in tumor cells because of the increased superoxide dismutase activity of these cells compared with healthy cells [113].

### Inhibition of invasion and metastasis

Gyps suppressed the invasion and migration of SCC4 human tongue cancer cells in a dose- and time-dependent manner by downregulating nuclear factor kappa B (NF-κB) and matrix metalloproteinase-9 (MMP-9) [114]. Gyps also inhibited the invasion and migration of SAS cells, as demonstrated by the results of in vitro wound-healing and Boyden Chamber assays. Treatment with Gyps led to decreases in the levels of several migration- and invasion-associated proteins, including NF-κB, cyclooxygenase-2, extracellular signal-regulated kinase 1/2 (ERK1/2), matrix metalloproteinase-2 (MMP-2), MMP-9, sevenless homolog, Ras, urokinase-type plasminogen activator, focal adhesion kinase and alpha serine/threonine protein kinase [115]. Furthermore, Gyps exhibited anti-migration activities towards SW620 human colon adenocarcinoma cells and Eca-109 human esophageal squamous carcinoma cells [19]. Gyps also inhibited the migration of SW-480 human colon adenocarcinoma cells in vitro at a concentration of 100 μg/mL [36]. This effect was observed in clinical studies. For example, patients with advanced malignant tumors that were treated with GpM formula showed a reduced cancer metastasis rate of 8.5 % compared with 55.2 % in the control group [36].

### Glycolysis inhibition

One of the hallmarks of cancer cells is deregulated energy metabolism, which can lead to a state known as ‘‘aerobic glycolysis’’ [116]. Targeting glucose metabolism has therefore proven to be a promising avenue for the development of new cancer treatments [117]. GpMix, which is a mixture of triterpenoid saponins from GpM, effectively inhibited the growth of cancer cells in the presence of co-cultivated normal cells [118]. Furthermore, GpMix exhibited both chemopreventive and therapeutic effects towards the formation of intestinal polyps in Apc^min/+^ mice (a mouse model of colon cancer). Several key enzymes along the glycolysis pathway, including pyruvate kinase (PK), α-enolase, glyceraldehyde 3-phosphate dehydrogenase (GAPDH), mitochondrial aconitase and ATP synthase-α and -β were found to be downregulated in R6 cells treated with GpMix by proteomic analysis [118]. These findings therefore implied that the inhibition of the glycolysis pathway was involved in the suppression of cell proliferation by GpMix.

### Immune modulation

GpM also exhibited anti-cancer effects indirectly through its immunomodulating activities. For example, Yang et al. [119] found that a water-soluble polysaccharide from *G. pentaphyllum* herb tea (PSGP) indirectly exerted anti-cancer activity against SW-1116 human colorectal adenocarcinoma cells and HT-29 by enhancing the immune response of macrophages with increased TNF-α secretion in a dose-dependent manner. Moreover, GP-B1, the acidic polysaccharide obtained from GpM, not only significantly inhibited the growth of cancer cells, but also improved cellular immune response with increased levels of TNF-α, IFN-γ, IL-10 and IL-12 in the serum of melanoma-B16-bearing mice [88]. The anti-cancer activity of Gyps was attributed to the elevated immune systems of the xenografted mice [120]. Gyps significantly suppressed tumor growth in mice transplanted with Lewis lung cancer cells with tumor weight inhibition rates of 29.8 ± 1.3, 51.4 ± 2.2 and 50.0 ± 1.6 % following intraperitoneal Gyps injections of 10, 20 and 40 mg/kg, respectively. Notably, the immune responses of these mice improved considerably, as demonstrated by increases in their total splenic cell number and the enhanced biological activities of the NK and splenic cells [120]. Clinical studies have also shown that GpM enhanced the activity of NK cells in breast cancer patients [100], improved the immune function of cancer patients after chemotherapy, increased the T lymphocyte transformation rate and decreased the IgG and IgM levels [101].

### Perspectives

Numerous studies have been published during the last four decades regarding the anti-cancer effects of GpM, including reports focused on (i) the isolation and characterization of its chemical components [121, 122]; (ii) the evaluation of its anti-cancer activities and mechanisms of action [107, 115]; and (iii) studies on its toxicity [96]. Taken together, the results of these reports have demonstrated that GpM has a broad anti-cancer spectrum (against 30 cancer cell lines, Table 3) without any obvious inhibitory effect on normal cell proliferation. However, there are limitations associated with most of these studies.

### The standard preparation of Gyps needs to be unified

Gyps consist of a mixture of approximately 189 dammarane-type saponin glycosides. Most of the studies reported to date on GpM have focused exclusively on the use of its fractions, such as Gyps, as well as the use of its extracts. In contrast, there have been very few reports pertaining to the use of single compounds isolated from GpM. For example, in all of the papers published during the last 15 years regarding the anti-cancer mechanisms of GpM there has only been one study involving the use of single compounds. Based on to the lack of chemical consistency in the fractions and extracts of GpM, greater efforts should be taken to explore the anti-cancer activities of single compounds derived from GpM in future studies, where possible. Moreover, none of the Gyps tested in any of the studies reported to date were prepared using a unified procedure, which could have led to completely different chemical component profiles amongst the different samples. Most of these studies also failed to provide essential chemical composition information for their Gyps, such as the exact molecular structure of each saponin, the number of saponins in the mixture, the relative contents of the different saponins in Gyps and a standard HPLC fingerprint [19, 89, 90, 106, 107, 109, 111, 115]. Studies on the chemical structures of the saponins in Gyps are therefore urgently needed, as well as further studies towards the chemical composition and the quantitative analysis of Gyps. These data would allow researchers to develop a deeper understanding of the anti-cancer activities and mechanisms of action of Gyps and facilitate further studies.

### Experimental systems need to be closer to the clinical settings

Most of the studies reported to date concerning the anti-cancer activities and mechanisms of action of GpM have been conducted using in vitro cellular systems. This trend could therefore explain why non-specific cell cycle arrest and the induction of apoptosis have been cited, in the majority of cases, as the principal mechanisms of action of GpM [89, 104, 106], with very few reports citing specific molecular targets or enzymatic pathways. In contrast, most of the in vivo studies conducted on GpM, have focused on the use of cancer cell lines implanted into immunodeficient mice [20, 90, 91]. According to this model, cancer cell lines are selected to survive in culture, and tumor-resident cells and proteins that interact with the cancer cells are eliminated to give a phenotypically homogeneous culture [123]. Patient-derived tumor xenograft (PDTX) models have several advantages over cell line xenograft models, such as maintaining the heterogeneity of the tumor and mimicking the microenvironment of human tumors [124]. Humanized-xenograft models can also be created by co-engrafting a sample of a patient-derived tumor together with peripheral blood or bone marrow cells into an immunodeficient mouse, followed by the reconstitution of the murine immune system. Advanced tumor models of this type can be used to study the interactions between xenogenic human stroma and tumor environments in cancer progression and metastasis [125]. Genetically engineered mouse models represent an interesting alternative for evaluating the effects of anti-cancer agents because these animals maintain a competent immune system, allowing for changes in the tumor microenvironment and the tumor itself to be thoroughly evaluated from an early stage [126]. Based on our review of the literature, we believe that further experiments should be performed in a PDTX, humanized-xenograft or genetically engineered mouse model to evaluate the effects of GpM on tumor development with greater clinical accuracy. The latter of these two models would be especially interesting in terms of evaluating the potential immunomodulatory activity of GpM.

## Conclusion

In summary, GpM has been investigated extensively as a potent anti-cancer agent against many types of cancers both in vitro and in vivo. The general consensus from the literature is that GpM exerts its anti-cancer activities through multiple mechanisms, including cell cycle arrest, the induction of apoptosis, inhibition of invasion and metastasis, glycolysis inhibition and immunomodulation.

## Additional file

10.1186/s13020-016-0114-9 Additional tables.

## Abbreviations

AIF: apoptosis inducing factor
Akt: alpha serine/threonine protein kinase
ANAE+: acid α-naphthyl acetate esterase
ATF4-α: activating transcription factor 4 alpha
ATF6-α: activating transcription factor 6 alpha
ATM: ataxia telangiectasia mutated
ATR: ataxia-telangiectasia and Rad3-related
CDK2: cyclin-dependent kinase 2
CDK4: cyclin-dependent kinase 4
CDK6: cyclin-dependent kinase 6
Chk2: checkpoint kinase 2
CM: Chinese medicine
COX-2: cyclooxygenase-2
DNAPK: DNA-dependent serine/threonine protein kinase
EndoG: endonuclease G
ER: endoplasmic reticulum
ERK1/2: extracellular signal-regulated kinase ½
FAK: focal adhesion kinase
GADD153: growth arrest and DNA damage-inducible gene 153
GpM: *Gynostemma pentaphyllum* (Thunb.) Makino
GAPDH: glyceraldehydes 3-phosphate dehydrogenase
GRP78: glucose-regulated protein 78
Gyps: gypenosides
IFN-γ: interferon-γ
IL-10: interleukin-10
IL-12: interleukin-12
MMP-2: matrix metalloproteinase-2
MMP-9: matrix metalloproteinase-9
NF-kB: nuclear factor kappa B
NK: natural killer
CTL: cytotoxic T lymphocytes
PK: pyruvate kinase
SOD: superoxide dismutase
SOS: sevenless homolog
TNA: tumor necrosis area
TNF-α: tumor necrosis factor-α
TTA: tumor total area
uPA: urokinase-type plasminogen activator
