# Supplementary material for: Anti-cancer effects of Gynostemma pentaphyllum (Thunb.) Makino (Jiaogulan)
Source: Chin Med. 2016 Sep 27;11:43. doi: 10.1186/s13020-016-0114-9 (PMC5037898; doi:10.1186/s13020-016-0114-9)
Supplement: Supplementary file 1 — 10.1186/s13020-016-0114-9 Additional tables. [file 13020_2016_114_MOESM1_ESM.docx]

Supplemental Information

Supplemental Table 1: Chemical structures of identified sterols from GpM

| 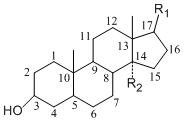 | | | | |
| --- | --- | --- | --- | --- |
| Compd | **R_1_** | **R_2_** | **Double bond(s)** | **Reference** |
| (24S)-5α-cholesta-5,22-dien-3β-ol | 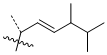 | H | C5-C6 | [[1](#_ENREF_1)] |
| (24R)-5-cholesta-5,22-dien-3β-ol | 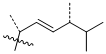 | H | C5-C6 | [[1](#_ENREF_1)] |
| β-sitosterol | 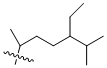 | H | C5-C6 | [[2](#_ENREF_2)] |
| 24,24-dimethyl-5-cholestan-3β-ol | 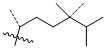 | H | ― | [[3](#_ENREF_3)] |
| 24α/R-ethyl-5α-cholestan-3β-ol | 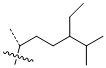 | H | ― | [[3](#_ENREF_3)] |
| (24S)-14-methyl-5α-ergost-9(11)-en-3β-ol | 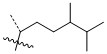 | H | C9-C11 | [[4](#_ENREF_4)] |
| (24R)-14α-methyl-5α-ergost-9(11)-en-3β-ol | 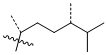 | H | C9-C11 | [[4](#_ENREF_4)] |
| 14α-methyl-5α-ergosta-9(11),24(18)-dien-3β-ol | 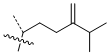 | H | C9-C11 | [[5](#_ENREF_5)] |
| 24,24-dimethyl-5α-cholesta-7-en-3β-ol | 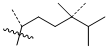 | H | C7-C8 | [[6](#_ENREF_6)] |
| (24E)-24,24-dimethyl-5α-cholesta-7,22-dien-3β-ol | 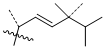 | H | C7-C8 | [[6](#_ENREF_6)] |
| 24,24-dimethyl-5α-cholesta-7,25-dien-3β-ol | 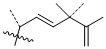 | H | C7-C8 | [[6](#_ENREF_6)] |
| (24R)-5α-stigmast-7-en-22-yn-3β-ol | 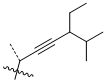 | CH_3_ | C7-C8 | [[7](#_ENREF_7)] |
| 24,24-dimethyl-5α-cholest-7-en-22-yn-3β-ol | 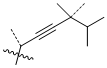 | CH_3_ | C7-C8 | [[7](#_ENREF_7)] |
| 24,24-dimethyl-5α-cholest-7,25-dien-22-yn-3β-ol | 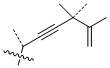 | CH_3_ | C7-C8 | [[7](#_ENREF_7)] |
| (24E,24R)-5α-stigmasta-7,22-dien-3β-ol | 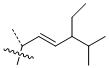 | H | C7-C8 | [[8](#_ENREF_8)] |
| Spinasterol | 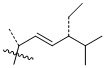 | H | C7-C8, C9-C11 | [[8](#_ENREF_8)] |
| Isofucosterol | 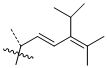 | H | C7-C8, C9-C11 | [[2](#_ENREF_2)] |
| 4α,14α-dimethyl-5α-ergosta-7,9(11),24(28)-trien-3β-ol | 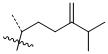 | CH_3_ | C7-C8, C9-C11 | [[9](#_ENREF_9)] |

Supplemental Table 2: Chemical structures of identified flavonoids from GpM

|  | | | | | | |
| --- | --- | --- | --- | --- | --- | --- |
| **Compd** | **R_1_** | **R_2_** | **R_3_** | **R_4_** | **R_5_** | **Reference** |
| Quercetin | H | H | H | OH | H | [[10](#_ENREF_10), [11](#_ENREF_11)] |
| Rutin | H | H | Rutinose | OH | H | [[10](#_ENREF_10), [11](#_ENREF_11)] |
| Ombuoside | CH_3_ | H | Rutinose | OH | CH_3_ | [[10](#_ENREF_10), [11](#_ENREF_11)] |
| Ombuin | CH_3_ | H | H | OH | CH_3_ | [[12](#_ENREF_12)] |
| Isorhamnetin-3-O-rutinoside | H | H | Rutinose | OCH_3_ | H | [[13](#_ENREF_13)] |
| Isorhamnetin | H | H | H | OCH_3_ | H | [[13](#_ENREF_13)] |
| Quercetin-di-(rhamno)-hexoside | H | Rhamnose | Rhamnose | OH | H | [[14](#_ENREF_14)] |
| Quercetin-rhamno-hexoside | H | H | Hexose-Rha | OH | H | [[14](#_ENREF_14), [15](#_ENREF_15)] |
| Kaempferol-rhamno-hexoside | H | H | Hexose-Rha | H | H | [[14](#_ENREF_14), [15](#_ENREF_15)] |
| Kaempferol-3-O-rutinoside | H | H | Rutinose | H | H | [[14](#_ENREF_14)] |

**References**

1. Akihisa T, Ghosh P, Thakur S, Rosentein F, Matsumoto T. Sterol compositions of seeds and mature plants of family cucurbitaceae. J Am Oil Chem Soc. 1986;63(5):653-8. doi:10.1007/BF02638231.

2. Marino A Fau - Elberti MG, Elberti Mg Fau - Cataldo A, Cataldo A. Sterols from Gynostemma pentafillum. (0037-8771 (Print)).

3. Akihisa T, Mihara H, Fujikawa T, Tamura T, Matsumoto T. 24, 24-Dimethyl-5α-cholestan-3β-ol, a sterol from Gynostemma pentaphyllum. Phytochemistry. 1988;27(9):2931-3.

4. Akihisa T, Kanari M, Tamura T, Matsumoto T. (24R)-and (24S)-14α-methyl-5α-ergost-9 (11)-en-3β-ols from Gynostemma pentaphyllum. Phytochemistry. 1989;28(4):1271-3.

5. Akihisa T, Tamura T, Matsumoto T. 14α-methyl-5α-ergosta-9 (11) 24 (28)-dien-3β-ol a sterol from Gynostemma pentaphyllum. Phytochemistry. 1987;26(8):2412-3: 0031-9422.

6. Akihisa T, Shimizu N, Tamura T, Matsumoto T. Structures of three new 24, 24-dimethyl-Δ7-sterols fromGynostemma pentaphyllumfromGynostemma pentaphyllum. Lipids. 1986;21(8):515-7: 0024-4201.

7. Akihisa T, Tamura T, Matsumoto T, Kokke W, Yokota T. Isolation of acetylenic sterols from a higher plant. Further evidence that marine sterols are not unique. The Journal of Organic Chemistry. 1989;54(3):606-10: 0022-3263.

8. Akihisa T, Thakur S, Rosenstein FU, Matsumoto T. Sterols of cucurbitaceae: The configurations at C-24 of 24-Alkyl-Δ5-, Δ7-and Δ8-sterols. Lipids. 1986;21(1):39-47: 0024-4201.

9. Akihisa T, Kokke W, Yokota T, Tamura T, Matsumoto T. 4α, 14α-Dimethyl-5α-ergosta-7, 9 (11), 24 (28)-trien-3β-ol from Phaseolus vulgaris and Gynostemma pentaphyllum. Phytochemistry. 1990;29(5):1647-51.

10. Yin F, Hu L, Lou F, Pan R. Dammarane-type glycosides from Gynostemma pentaphyllum. Journal of natural products. 2004;67(6):942-52.

11. Fang ZP, Zeng XY. Isolation and identification of flavonoids and organic acids from Gynostemma pentaphyllum Makino. Zhongguo Zhong yao za zhi = Zhongguo zhongyao zazhi = China journal of Chinese materia medica. 1989;14(11):676-8, 703.

12. Zha-Pu F, Xian-Yi Z. Isolation and identification of flavonoid glycosides and organic acid from *Gynostemma pentaphyllum*(Thunb.) Makino. China Journal of Chinese Materia Medica. 1989;14(11):36.

13. Qi C, Di-Hua C, Lian-Gang S, Jian-Yong S, Jian-Qiang Z, Xue-Lan L. Chmical composition Study on Gynstemma pubescens (Gagnep.) C. Y. Wu—Flavonoids. Natural Product Research and Development. 1991;4:002.

14. Kao T, Huang S, Inbaraj BS, Chen B. Determination of flavonoids and saponins in Gynostemma pentaphyllum (Thunb.) Makino by liquid chromatography–mass spectrometry. Analytica chimica acta. 2008;626(2):200-11.

15. Tiberti LA, Yariwake Jh Fau - Ndjoko K, Ndjoko K Fau - Hostettmann K, Hostettmann K. Identification of flavonols in leaves of Maytenus ilicifolia and M. aquifolium (Celastraceae) by LC/UV/MS analysis. (1570-0232 (Print)).
